# Preparation of p-Methoxy-m-Nitrobenzoic Acid via Catalytic Oxidation Method in Water Solvent

**DOI:** 10.3390/molecules31101766

**Published:** 2026-05-21

**Authors:** Guohang Zhuang, Liuye Mo, Iemasa Yao

**Affiliations:** 1 Zhejiang Key Laboratory of Pollution Control for Port-Petrochemical Industry, School of Petroleum Chemical Engineering & Environment, Zhejiang Ocean University, Zhoushan 316022, China; zhuangguohang@126.com; 2 Zhoushan Olichem Chemical Co., Ltd., Zhoushan 316054, China; jiachang0507@126.com

**Keywords:** water solvent, catalytic oxidation, p-methoxy-m-nitrobenzyl alcohol (MMNA), p-methoxy-m-nitrobenzoic acid (MNBA), TEMPO/KBr/NaClO_2_ system

## Abstract

p-Methoxy-m-nitrobenzoic acid (MNBA) serves as a valuable chemical intermediate across numerous domains. Nevertheless, the synthesis of MNBA through non-catalytic oxidation processes invariably results in the production of environmentally polluting substances. In this study, we report an environmentally benign catalytic oxidation system for the synthesis of MNBA using water as a solvent. Based on the two-step TEMPO/KBr/NaOCl/NaClO_2_ system, which achieved a 91.4% yield at 70 °C, we have devised a simplified one-step procedure employing the TEMPO/KBr/NaClO_2_ system. This less energy-intensive input method yields 90.1% MNBA at 60 °C. Systematic optimization has revealed that temperature, time, and oxidant quantity are critical parameters. Furthermore, acidic conditions have been found to reduce yields due to the decomposition of NaClO_2_. The aqueous-phase approach completely avoids organic solvents and facilitates product isolation. A synergistic catalytic mechanism involving N-oxoammonium intermediates is proposed. This work establishes a sustainable strategy for preparing multifunctional aromatic carboxylic acids, addressing key challenges in both ecological impact and industrial scalability for fine chemical production.

## 1. Introduction

Organic carboxylic acids, as ubiquitous structural motifs, have traditionally been synthesized via the oxidation of alcohols or aldehydes [1]. Alternative methodologies, such as nitrile hydrolysis and organometallic compound carbonation, are indeed available [2,3,4]; however, oxidative pathways continue to be the primary methods employed. Conventional stoichiometric oxidation methods employ environmentally hazardous reagents such as KMnO_4_ [5], K_2_Cr_2_O_7_ [6], and hypervalent iodine compounds [7]. These oxidants generate substantial waste and complicate product isolation procedures. Therefore, the development of environmentally friendly catalytic oxidation methods is urgently needed. Karthikeyan et al. [8] demonstrated an efficient catalytic oxidation system using CuCl_2_/BIL (bifunctional ionic liquid) and 30% H_2_O_2_, and it directly converted aliphatic and aromatic primary alcohols to carboxylic acids under solvent-free conditions at 25 °C with high yields (84–99%). The catalyst exhibited remarkable stability, retaining full activity over five reuse cycles. Ahmed et al. [9] developed a robust Pd-Bi-Te/C catalyst system for the aerobic oxidation of diverse primary alcohols to carboxylic acids using O_2_ in a H_2_O/MeOH solvent system at 50 °C, achieving high yields (up to >99%). The catalyst demonstrated excellent stability in continuous flow, exceeding 30,000 turnovers. Nejad et al. [10] designed a magnetically recoverable Cu (II) catalyst (Fe_3_O_4_@L-Arg-CD-Cu (II)) that catalyzed efficient TBHP oxidation of primary alcohols and benzyl halides to carboxylic acids (≤92% yield) under solvent-free conditions at 90 °C, with five-fold recyclability and minimal metal leaching (0.34% Cu). Recently, Chen et al. [11] developed a magnetically recoverable Co@NC catalyst for the acceptorless dehydrogenation of primary alcohols to carboxylic acids using KOH in refluxing mesitylene, achieving yields up to 95% and excellent recyclability over 15 cycles without significant activity loss. Additionally, Yu et al. [12] developed an amphiphilic lacunary polyoxometalate catalyst, [(CH_3_)_3_NC_12_H_25_]_2_Na_5_PW_11_O_39_, for oxidizing primary alcohols to carboxylic acids using H_2_O_2_ as the oxidant. This system achieved 80% conversion of 1-propanol with 73% selectivity to propionic acid. It also showed a broad substrate scope and maintained good performance over four cycles.

**Scheme 1:**
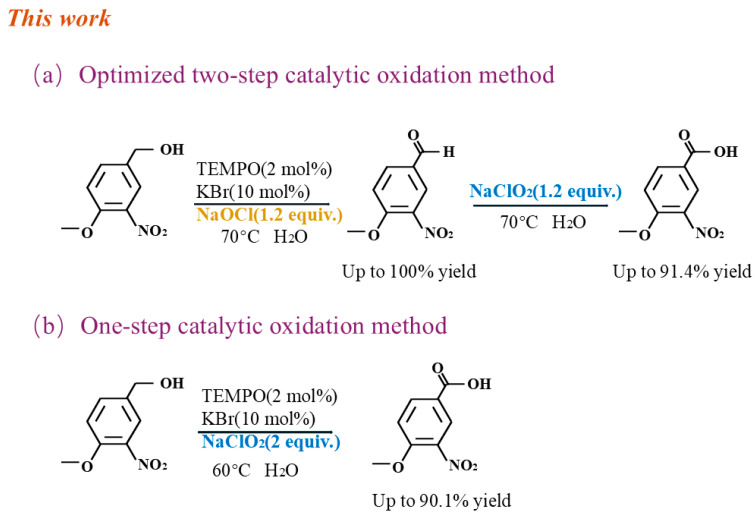
Catalytic preparation methods of MNBA.

Although significant progress has been achieved in the catalytic oxidation of alcohols to carboxylic acids, limitations persist regarding environmental issues, energy efficiency, cost-effectiveness, and process scalability. Developing new catalytic oxidation systems is particularly important. Noticeably, 2,2,6,6-Tetramethylpiperidine-1-oxyl (TEMPO), a stable nitroxyl radical [13,14], has emerged as an attractive catalyst due to its excellent oxidation selectivity and environmentally benign characteristics. Ma’s research group [15] reported the first instance of copper-catalyzed aerobic oxidation of primary alcohols to carboxylic acids, using TEMPO and KHSO_4_ as co-catalysts and O_2_ as the oxidant under mild conditions. The method exhibited broad substrate compatibility, including chiral and functionalized alcohols, and afforded the acids in high yields without racemization. Limitations include prolonged reaction times (36–72 h) and the small scale of standard reactions (0.5–1.0 mmol). Zhao et al. [16,17] modified Anelli’s TEMPO/KBr/NaOCl-catalyzed oxidation procedure [18] by employing NaClO_2_ as the primary oxidant with catalytic TEMPO/NaOCl under milder conditions (pH 6.7, 35 °C). This adjustment effectively suppressed chlorination side reactions and enabled a stereospecific one-pot conversion of alcohols to carboxylic acids. However, the method remained ineffective for allylic and electron-rich benzylic alcohols. Zanka [19] improved Zhao’s method by separating the one-pot oxidation into two steps—TEMPO/KBr/NaOCl oxidation at pH 8–10 to form the aldehyde, followed by NaClO_2_ oxidation at pH 5.0 to the acid. This enhanced process safety and scalability by preventing unstable NaClO/NaClO_2_ mixing and improving reaction control. A significant constraint is its incompatibility with electron-rich aromatic substrates, such as p- and m-methoxybenzyl alcohols.

p-Methoxy-m-nitrobenzoic acid (MNBA) represents a valuable scaffold in medicinal chemistry and dye manufacturing [20,21,22]. It also shows considerable potential in plant physiology studies, antiviral drug synthesis, and engineered enzyme evaluation [23,24,25]. Although important advancements have recently been made in catalytic methods for synthesizing organic carboxylic acids, catalytic oxidation synthesis of MNBA remains challenging owing to steric and electronic effects imposed by its multisubstituted aromatic ring. Current synthesis methods still rely on traditional non-catalytic oxidation approaches. Based on this, this study focuses on MNBA as the target compound, exploring its synthesis via the catalytic oxidation of p-methoxy-m-nitrobenzyl alcohol (MMNA). MMNA can be easily obtained by hydrolyzing p-methoxy-m-nitrobenzyl chloride (MNBC). As a precursor to MMNA, MNBC can itself be efficiently prepared through the chloromethylation of o-nitroanisole [26]. Therefore, in this paper, we selected inexpensive and readily available MMNA as the starting material to synthesize MNBA via a catalytic oxidation pathway. Through method optimization, we developed a simplified one-step catalytic oxidation protocol (Scheme 1b) that offers comparable yield and superior operational simplicity compared to the two-step approach (Scheme 1a).

## 2. Materials and Methods

The chemicals and characterization instruments used in this work are listed in the Supplementary Materials. MMNA (obtained from heated hydrolysis) and MNBA (obtained from the experiment) were characterized by FT-IR, ^1^H NMR, and melting point measurement. The intermediate (p-methoxy-m-nitrobenzaldehyde) and byproduct (o-nitroanisole) were isolated and identified by FT-IR and ^1^H NMR. The analysis data are presented in the Supplementary Materials (Figures S1–S10, Tables S1–S4).

### 2.1. Procedure for p-Methoxy-m-Nitrobenzyl Alcohol (MMNA) Preparation

MMNA was synthesized in almost quantitative yield by the thermal hydrolysis of p-methoxy-m-nitrobenzyl chloride (MNBC). A suspension of MNBC (10.0 g, 49.6 mmol) in deionized water (300 mL) was heated at 90 °C for 3 h in a 500 mL round-bottom flask fitted with a condenser. After the reaction was complete, the resulting mixture was concentrated at 60 °C, affording MMNA as yellow crystals (8.90 g, 98% yield).

### 2.2. General Procedure for Optimized Two-Step Catalytic Oxidation Method

The two-step catalytic oxidation method is depicted in Scheme 1a. In a 250 mL round-bottom flask equipped with a condenser, MMNA (5.00 g, 27.0 mmol), TEMPO (0.08 g, 0.55 mmol), and KBr (0.32 g, 2.70 mmol) were put into deionized water (40 mL). After temperature stabilization at a set point, NaOCl solution (25 mL, 33 mmol) was added dropwise over 30 min, followed by stirring for an additional 30 min. The pH was then carefully adjusted to 5.0 using aqueous HCl (35 wt%). Subsequently, NaClO_2_ (33 mmol) was dissolved in deionized water (40 mL) before being added dropwise over 30 min, and the mixture was stirred for 3 h (with hourly TLC monitoring, Figures S11–S16). Upon completion of the reaction, the mixture was cooled to 25 °C, and aqueous NaOH (10 mL, 40 mmol) was added to form the sodium carboxylate. After being stirred for 30 min and allowed to stand for 15 min, the mixture was vacuum-filtered. The filtrate was acidified to yield MNBA by simple filtration.

### 2.3. General Procedure for One-Step Catalytic Oxidation Method

The one-step catalytic oxidation method is depicted in Scheme 1b. In a 250 mL round-bottom flask fitted with a condenser, MMNA (5.00 g, 27.0 mmol), TEMPO (0.08 g, 0.55 mmol), and KBr (0.32 g, 2.70 mmol) were put into deionized water (40 mL). Upon reaching a stable temperature, NaClO_2_ (66 mmol) was dissolved in deionized water (40 mL) before being added dropwise over 50 min under vigorous stirring, and the reaction mixture was stirred for 3 h (monitored by TLC, Figures S17–S21). After completion of the reaction, the MNBA product was separated as described in Section 2.2.

CAUTION: Although NaClO_2_ is commonly used in oxidation reactions, because it is a strong oxidant, the reaction must be controlled in a safe way. Therefore, the reaction temperature should not be higher than 80 °C. Furthermore, the NaClO_2_ should be added slowly. Otherwise, the reaction may be intense and even cause an explosion.

## 3. Results

### 3.1. Investigation of the Two-Step Catalytic Oxidation Method for MNBA Synthesis

Based on the conventional two-step oxidation process (Scheme 1a), reaction parameters including solvent, temperature, time, and oxidant amount were systematically optimized, as shown in Table 1.

It can be seen from Table 1 that the solvents have a significant effect on the yield of MNBA at room temperature (Entries 1–3). The yields were 30.4% and 55.2% in ethyl acetate (EtOAc) and acetonitrile, respectively. Notably, under Zanka’s original conditions (EtOAc, room temperature), the yield was only 30.4% (Entry 1), confirming that MMNA exhibits poor reactivity, similar to other electron-rich benzyl alcohol substrates reported in the literature [19]. It is widely acknowledged that the use of water as a solvent is favored for environmental protection, while the employment of toxic organic solvents is generally eschewed in a reaction. Regrettably, the formation of trace amounts of MNBA in an aqueous solvent is likely attributable to the substrate’s low solubility in water at room temperature (Entry 3). In order to increase the MNBA yield, the reaction temperature was elevated above room temperature. The yield increased from trace amounts at room temperature to 77.5% at 60 °C (Entry 4), reaching a maximum of 91.4% at 70 °C (Entry 6). This dramatic improvement demonstrates that, by employing water as the solvent and elevating the reaction temperature, the limitations previously associated with TEMPO oxidation of electron-rich aromatic alcohols [19] can be effectively overcome. The optimal yield of 91.4% was achieved at 70 °C (Entry 6). Beyond 70 °C, the yield decreases to 86.1% (Entry 8), owing to decarboxylation at elevated temperatures.

Reaction time was also optimized at 70 °C. The yield reached 84.0% at 2 h and 91.4% at 3 h. Prolonging to 4 h decreased the yield to 82.3% due to decarboxylation. Our results are consistent with the literature [19]. The oxidant amount and type also critically affect reaction outcomes. When using 2.4 equivalents of NaOCl (Entry 9), the reaction is arrested at the aldehyde intermediate without progressing to carboxylic acid formation, yielding only trace amounts of MNBA. Interestingly, when the NaOCl step was omitted (Entry 10), an 81.1% yield was still obtained, demonstrating that NaClO_2_ alone, with TEMPO and KBr catalysts, can oxidize the MMNA directly to the MNBA. Control tests (Entries 11–12) confirm the necessity of all three components (TEMPO/KBr/oxidant), with trace conversion observed when any component is omitted. This optimized aqueous-phase system achieved high efficiency without organic solvents while maintaining exceptional operational simplicity and minimal environmental impact, all of which are key advantages for industrial-scale applications.

### 3.2. Investigation of One-Step Catalytic Oxidation Method for MNBA Synthesis

The experimental results in Section 3.1 confirm that NaClO_2_ serves as the sole terminal oxidant in the TEMPO/KBr/NaClO_2_ catalytic system, effectively converting the substrate to the target carboxylic acid in water solvent. Therefore, we systematically investigated the TEMPO/KBr/NaClO_2_ catalytic system, with particular emphasis on key parameters such as reaction temperature, oxidant amount, time, and pH (Table 2). Evidently, temperature exhibited the most significant impact on reaction outcomes. At 60 °C, the yield reaches a maximum of 85.6% (Entry 8). Below this temperature, the MNBA is only afforded in trace amounts as the substrate shows low solubility in the reaction mixture (Entry 1). Nevertheless, above 60 °C, the yields gradually decline to 81.1% at 70 °C (Entry 9) and 79.1% at 80 °C (Entry 10), owing to decarboxylation. The amount of oxidant is not an influencing factor on the MNBA yield, as the addition of NaClO_2_ is higher than 1.6, although it is still an important parameter. At 60 °C, 1.6 equivalents of sodium chlorite yield 89.3% (Entry 2), comparable to the yield obtained with 2.0 equivalents (Entry 4, 90.1%). However, excess oxidant (>2.0 equivalents) promotes decarboxylation, thereby lowering the yield (Entry 8). From an industrial perspective, using 1.6 equivalents of sodium chlorite proves more cost-effective. Reaction time was optimized at 60 °C with 2.0 equivalents of NaClO_2_. The yield reaches 82.4% after 2 h (Entry 3) and increases to 90.1% at 3 h (Entry 4). A further extension to 4 h leads to a slight decline to 83.2% (Entry 5). Thus a reaction time of 3 h was identified as the optimal reaction time.

The pH of the reaction system also plays a pivotal role. Adding HCl to increase acidity reduces the yield to 44.9% (Entry 6), far below the 90.1% achieved under unadjusted conditions (Entry 4). This drop arises from accelerated NaClO_2_ decomposition in acidic media, narrowing the catalytic oxidation operation window [19]. The stability of the oxidant was also examined by stirring the reaction mixture for 3 h at 60 °C prior to the addition of the MMNA substrate. Regarding Entry 7, it was found that the yield of the target product still stayed at a high level (83.5%), showing that the oxidant of NaClO_2_ is stable in the absence of MMNA. A control experiment without TEMPO (Entry 11) afforded only trace amounts of the target product, verifying that KBr alone is incapable of catalyzing the reaction and that it acts merely as a co-catalyst. As shown in Table 2, the catalytic activity relies on the synergistic combination of TEMPO, KBr, and NaClO_2_. The removal of any component significantly reduces the yield (Entry 12).

A two-fold scale-up experiment employing 10.00 g of MMNA was carried out under the optimized conditions to assess safety and reproducibility. During the slow addition of NaClO_2_ solution, the reaction proceeded smoothly with mild exothermicity (temperature rising <5 °C), and no violent reaction or safety risks were detected. This scale-up trial produced MNBA in 88.7% yield (Entry 13), clearly demonstrating the reliability, safety, and scalability of the one-step protocol. Furthermore, the recyclability of the aqueous phase containing the TEMPO/KBr mixture was investigated. The aqueous catalyst layer was reused directly without supplementing additional TEMPO and KBr, except for the oxidant of NaClO_2_. As shown in Entries 14 and 15 of Table 2, the MNBA yields remained as high as 88.5% in the second run and 87.2% in the third run. The results indicate that the catalyst shows excellent reusability, which makes it highly desirable for industrial application. On the basis of the above results, a practical one-step catalytic oxidation system has been established for the synthesis of MNBA in aqueous media under mild conditions, featuring high efficiency and excellent selectivity. Based on these insights, we developed a practical one-step catalytic oxidation system for synthesis of MNBA under mild conditions in water solvent, achieving high efficiency and selectivity.

## 4. Reaction Mechanism

**Scheme 2:**
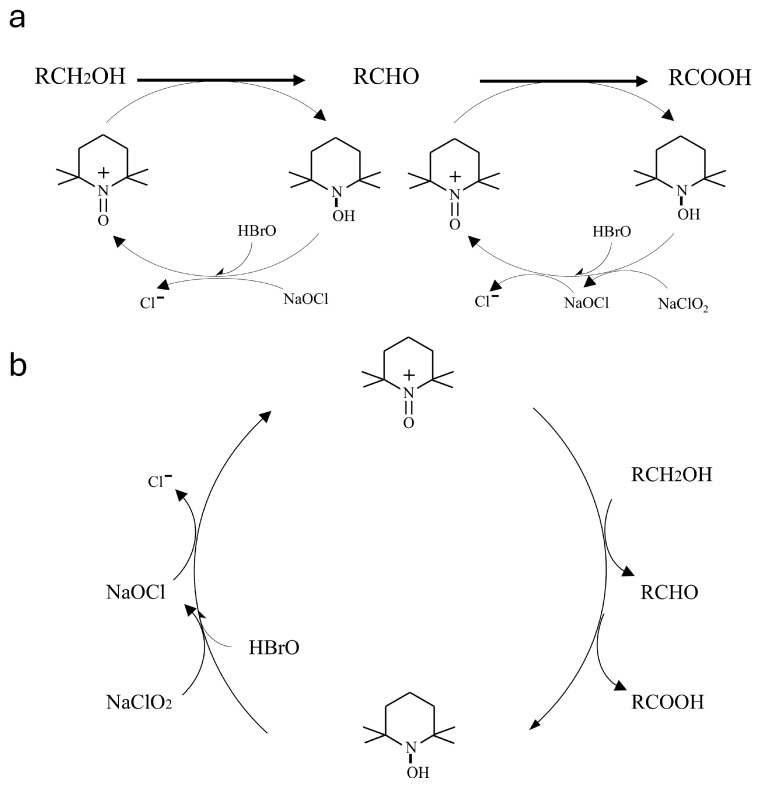
Proposed catalytic cycle for (**a**) the two-step catalytic oxidation method; (**b**) the one-step catalytic oxidation method.

The catalytic oxidation proceeds via two synergistic pathways (Scheme 2). In this system, TEMPO and KBr serve as catalysts, while NaOCl and NaClO_2_ act as terminal oxidants, a role assignment consistent with the classic TEMPO-catalyzed oxidation protocols established by Anelli et al. [18] and Zhao et al. [16,17].

In the two-step system (Scheme 2a), TEMPO is oxidized to the N-oxoammonium ion either directly by NaOCl or indirectly via HOBr formed through hypochlorite-induced bromide oxidation [27]. The N-oxoammonium species selectively oxidizes MMNA to the corresponding aldehyde [28]. Subsequently, NaClO_2_ reoxidizes TEMPO to regenerate the catalytic species while being reduced to NaOCl, which reactivates bromide oxidation. This regenerated N-oxoammonium species then further oxidizes the aldehyde intermediate to the carboxylic acid product [29]. Concurrently, the hydroxylamine intermediate either directly oxidizes to the N-oxoammonium species or undergoes comproportionation, generating TEMPO radicals that maintain catalytic cycling through reoxidation [30]. This cascade enables efficient alcohol-to-carboxylic acid conversion in two steps. The one-step system (Scheme 2b) utilizes NaClO_2_’s bifunctionality—TEMPO oxidation to N-oxoammonium species coupled with its reduction to NaOCl, which cyclically oxidizes bromide to regenerate active species. Integration of these steps creates a self-sustaining oxidant cycle upon initiation. Oxidant pathway optimization enhances overall system performance, practicality, and cost-efficiency.

## 5. Conclusions

In summary, this study optimized the existing two-step catalytic oxidation method, achieving a 91.4% yield of MNBA in the aqueous phase at 70 °C, and developed a one-step TEMPO/KBr/NaClO_2_ catalytic system, affording 90.1% yield in the aqueous phase at 60 °C. This water-based process, when operating under mild conditions, is environmentally friendly. Our approach provides an efficient and environmentally friendly route for MNBA synthesis, addressing a knowledge gap in catalytic oxidation while reducing environmental impact and production costs. This methodology provides an efficient approach for the environmentally benign oxidation of aromatic compounds which are difficult to oxidize through typical catalytic systems.

## Figures and Tables

**Table 1:** Two-step catalytic oxidation method for MNBA synthesis ^a^.

Entry	TEMPO (mol%)	KBr (mol%)	NaOCl (Equiv.)	NaClO_2_ (Equiv.)	Solvent	T (°C)	Yield ^d^ (%)
1	2	10	1.2	1.2	EtOAc	RT	30.4
2	2	10	1.2	1.2	CH_3_CN	RT	55.2
3	2	10	1.2	1.2	H_2_O	RT	Trace
4	2	10	1.2	1.2	H_2_O	60	77.5
5	2	10	1.2	1.2	H_2_O	70	84.0 ^b^
6	2	10	1.2	1.2	H_2_O	70	91.4
7	2	10	1.2	1.2	H_2_O	70	82.3 ^c^
8	2	10	1.2	1.2	H_2_O	80	86.1
9	2	10	2.4	-	H_2_O	70	Trace
10	2	10	-	2.4	H_2_O	70	81.1
11	-	-	1.2	1.2	H_2_O	70	Trace
12	2	10	-	-	H_2_O	70	Trace

^a^ the reaction was performed using MMNA (5.00 g, 27.0 mmol), TEMPO (0.08 g, 0.55 mmol), and KBr (0.32 g, 2.70 mmol), which were mixed in solvent (40 mL). Sodium hypochlorite was added to initiate the reaction, followed by stirring for 30 min. The pH of the system was then adjusted to 5, after which sodium chlorite was added, and the reaction was allowed to proceed for 3 h. ^b^ after adding the sodium chlorite solution, the reaction was allowed to continue for 2 h. ^c^ after adding the sodium chlorite solution, the reaction was allowed to continue for 4 h. ^d^ for the optimal conditions (Entry 6, 70 °C, 3 h), three independent replicates gave a mean yield of 91.4% ± 0.3%.

**Table 2:** One-step catalytic oxidation for MNBA synthesis ^a^.

Entry	TEMPO (mol%)	KBr (mol%)	NaClO_2_ (Equiv.)	T (°C)	Yield ^f^ (%)
1	2	10	2.4	55	Trace
2	2	10	1.6	60	89.3
3	2	10	2.0	60	82.4 ^b^
4	2	10	2.0	60	90.1
5	2	10	2.0	60	83.2 ^c^
6	2	10	2.0	60	44.9 ^d^
7	2	10	2.0	60	83.5 ^e^
8	2	10	2.4	60	85.6
9	2	10	2.4	70	81.1
10	2	10	2.4	80	79.1
11	-	10	2.4	80	Trace
12	-	-	2.4	80	Trace
13	2	10	2.0	60	88.7 ^g^
14	2	10	2.0	60	88.5 ^h^
15	2	10	2.0	60	87.2 ^i^

^a^ the reaction was conducted using MMNA (5.00 g, 27.0 mmol), TEMPO (0.08 g, 0.55 mmol), and KBr (0.32 g, 2.70 mmol) in water (40 mL). NaClO_2_ solution (40 mL) was added dropwise over 50 min, followed by continuous stirring for 3 h. Under optimal conditions (Entry 4), the pH value rises from 6.0 to 7.2 after NaClO_2_ addition, before gradually decreasing to 5.3 as the reaction takes place. ^b^ reaction time of 2 h. ^c^ reaction time of 4 h. ^d^ for comparative studies, hydrochloric acid (3 mL) was added dropwise to the reaction system before oxidation to adjust the acidity. ^e^ after stirring TEMPO (0.08 g, 0.55 mmol), KBr (0.32 g, 2.70 mmol), deionized water (40 mL), and NaClO_2_ solution (40 mL, 66 mmol) at 60 °C for 3 h, MMNA was added, and the reaction continued for another 3 h. ^f^ For the optimal conditions (Entry 4, 60 °C, 3 h), three independent replicates gave a mean yield of 90.1% ± 0.3%. ^g^ The reaction was conducted using MMNA (10.00 g, 54.0 mmol), TEMPO (0.16 g, 1.10 mmol), and KBr (0.64 g, 5.40 mmol) in water (80 mL). NaClO_2_ solution (80 mL) was added dropwise over 100 min, and the mixture was stirred at 60 °C for 3 h. ^h^ the catalytic system and aqueous phase were reused for the second cycle without adding fresh TEMPO and KBr. ^i^ the catalytic system and aqueous phase were reused for the third cycle without adding fresh TEMPO and KBr.

## Data Availability

The datasets used and/or analyzed during the current study are available from the corresponding author on reasonable request.

## Supplementary Materials

The following supporting information can be downloaded at: https://www.mdpi.com/article/10.3390/molecules31101766/s1, Figure S1: TLC image of the hydrolysis product; Figure S2: TLC image of the synthesized product; Figure S3: FT-IR spectra of the hydrolysis product and the MMNA standard; Figure S4: FT-IR spectra of the synthesized product and the MNBA standard; Figure S5: ^1^H NMR analysis (DMSO-d_6_) of the hydrolysis product; Figure S6: ^1^H NMR analysis (DMSO-d_6_) of the synthesized product; Figure S7: FT-IR spectra of the isolated aldehyde intermediate and the standard; Figure S8: FT-IR spectra of the isolated byproduct; Figure S9: ^1^H NMR analysis (DMSO-d_6_) of the isolated aldehyde intermediate; Figure S10: ^1^H NMR analysis (DMSO-d_6_) of the isolated byproduct; Figure S11: TLC image of optimized two-step reaction mixture; Figure S12: TLC image after the addition of NaOCl for 30 min; Figure S13: TLC image after the addition of NaClO_2_ for 1h; Figure S14: TLC image after the addition of NaClO_2_ for 2 h; Figure S15: TLC image after the addition of NaClO_2_ for 3 h; Figure S16: TLC image after the addition of NaClO_2_ for 4 h; Figure S17: TLC image of one-step reaction mixture; Figure S18: TLC image of the one-step reaction for 1 h; Figure S19: TLC image of the one-step reaction for 2 h; Figure S20: TLC image of the one-step reaction for 3 h; Figure S21: TLC image of the one-step reaction for 4 h; Table S1: Melting point of the hydrolysis product; Table S2: Melting point of the MMNA standard; Table S3: Melting point of the synthesized products; Table S4: Melting point of the MNBA standard. References [31,32,33,34,35,36,37,38,39,40,41,42,43] are cited in the Supplementary Materials.

## Abbreviations

The following abbreviations are used in this manuscript:

MMNA	p-Methoxy-m-nitrobenzyl alcohol
MNBA	p-Methoxy-m-nitrobenzoic acid
MNBC	p-Methoxy-m-nitrobenzyl chloride
TEMPO	2,2,6,6-Tetramethylpiperidine-1-oxyl
