# Supplementary material for: Preparation of p-Methoxy-m-Nitrobenzoic Acid via Catalytic Oxidation Method in Water Solvent
Source: Molecules. 2026 May 21;31(10):1766. doi: 10.3390/molecules31101766 (PMC13210257; doi:10.3390/molecules31101766)
Supplement: Supplementary file 1 [file molecules-31-01766-s001.zip › molecules-4303096-supplementary.pdf]

Supporting information

Preparation of p-Methoxy-m-Nitrobenzoic Acid via Catalytic Oxidation Method in Water Solvent

Guohang Zhuang [1], Liuye Mo [1,*] and Iemasa Yao [2]

[1] Zhejiang Key Laboratory of Pollution Control for Port-Petrochemical Industry, School of Petroleum Chemical Engineering & Environment, Zhejiang Ocean University, Zhoushan 316022, China; zhuangguohang@126.com
[2] Zhoushan Olichem Chemical Co., Ltd., Zhoushan 316054, China; jiachang0507@126.com
* Correspondence: liuyemo@zjou.edu.cn

1 Materials and Apparatus

All reagents were used as received from commercial suppliers without further purification. p-Methoxy-m-nitrobenzyl chloride (MNBC, purity ≥98%) and p-methoxy-m-nitrobenzoic acid (MNBA, purity ≥98%) were supplied by Zhoushan Olichem Chemical Co., Ltd. (Zhoushan, China). p-Methoxy-m-nitrobenzaldehyde (purity ≥98%) was purchased from Shanghai Yuanye Bio-Technology Co., Ltd. (Shanghai, China). p-Methoxy-m-nitrobenzyl alcohol (MMNA, purity ≥97%), 2,2,6,6-Tetramethylpiperidine-1-oxyl (TEMPO, purity ≥98%), Potassium bromide (KBr, purity ≥99%), Sodium hypochlorite (NaOCl, 6% aqueous solution), Sodium chlorite ($NaClO_2$, purity ≥80%), Acetonitrile (CH_3CN, purity ≥99%), Petroleum ether (PE, bp 60-90 °C) and Ethyl acetate (EtOAc, purity ≥99%) were obtained from Shanghai Macklin Biochemical Co., Ltd. (Shanghai, China). Sodium hydroxide (NaOH, purity ≥98%) and Hydrochloric acid (HCl, purity ≥36%) were purchased from Sinopharm Group Co., Ltd. (Shanghai, China). Deionized water was used throughout all experiments.

FT-IR spectra (400–4000 cm^{-1}) were acquired on a Nicolet 6700 spectrometer using KBr pellets. Melting points were measured with an MP430 automated apparatus. Proton nuclear magnetic resonance (^1H NMR) was recorded at 500 MHz on a Bruker Avance spectrometer using DMSO-d_6 as the solvent.

2 Product characterization

2.1 Thin-layer chromatography analysis

The product (obtained from heated hydrolysis of MNBC) was monitored by thin-layer chromatography (TLC) with ethyl acetate and petroleum ether (1:1, v/v) as the eluent. Ethanol solutions of the hydrolysis product (Figure S1(3)) and reference standards were spotted onto the same TLC plate and co-developed. The developed plate was visualized under a UV lamp.

The calculated Rf values were 0.78 for the MNBC standard, 0.42 for the authentic MMNA, and 0.42 for the hydrolysis product. The hydrolysis product of MMNA and the authentic MMNA have same Rf values, which can be identified temporarily as the same compound. No additional spots were detected, confirming the high purity of the product.

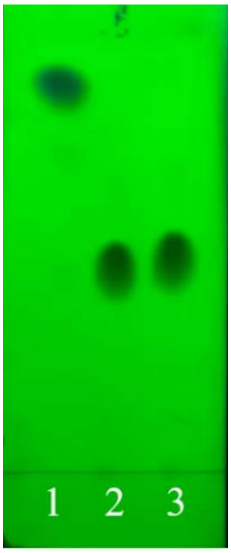

Figure S1. TLC image of the hydrolysis product.

(1) MNBC (standard), (2) MMNA (standard), (3) The hydrolysis product

While slight tailing occurred for the MNBA standard, effective separation was obtained between MNBA and its precursors (MMNA, Rf = 0.42; p-methoxy-m-nitrobenzaldehyde, Rf = 0.62). As shown in Figure S2, the developed TLC plate exhibited similar migration distances for both the synthesized product (Rf = 0.31) and the authentic MNBA standard (Rf = 0.31), with no extraneous spots observed. These results confirm the high purity of the synthesized MNBA.

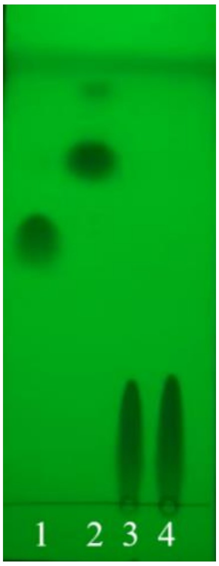

Figure S2. TLC image of the synthesized product.

(1) MMNA (standard), (2) p-Methoxy-m-nitrobenzaldehyde (standard),

(3) MNBA (standard), (4) The synthesized product

2.2 Determination by infrared spectroscopy

The hydrolysis product obtained from MNBC was blended with KBr and compressed into a pellet for Fourier-transform infrared (FT-IR) spectroscopic analysis. The acquired spectrum was then compared to that of an authentic MMNA standard, as described in Figure S3. The FT-IR spectrum of the hydrolysis product displayed characteristic absorption bands corresponding to key functional groups: aromatic ring vibrations at 1626 and 1526 cm^{-1} [31], nitro group stretching at 1353 cm^{-1} [32], meta-substitution of the benzene ring at 764 cm^{-1} [32], methoxy stretching at 1277 cm^{-1}, para-substitution at 822 cm^{-1} [33], a broad O–H stretching band at 3600–3200 cm^{-1}, and hydroxy C–O stretching at 1052 cm^{-1}. The close match between the two spectra confirmed the identity of the hydrolysis product as MMNA.

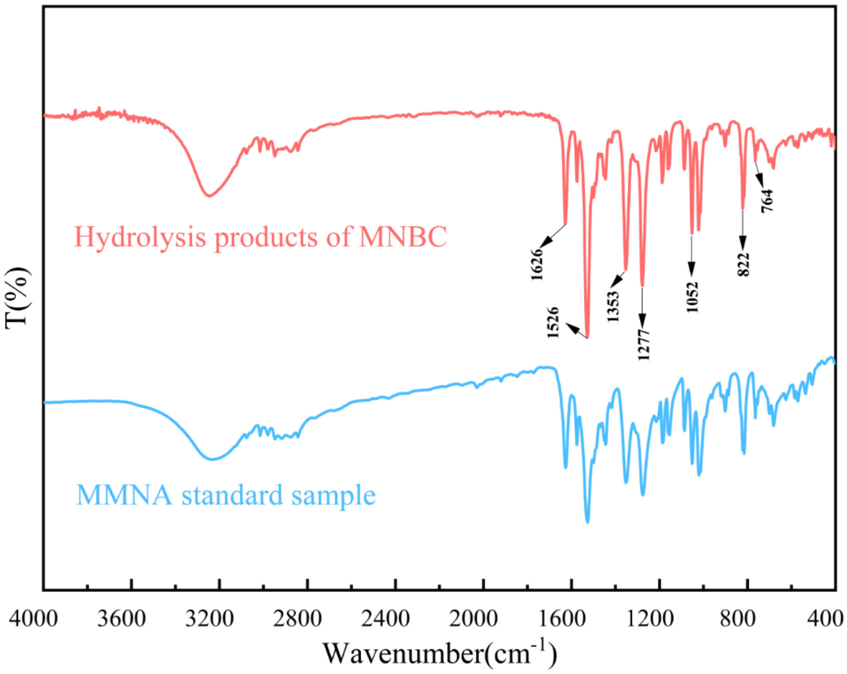

Figure S3. FT-IR spectra of the hydrolysis product and the MMNA standard.

The FT-IR spectrum of the synthesized product (prepared as a KBr pellet) exhibited excellent correlation with the authentic MNBA standard (Figure S4), displaying all characteristic absorption bands: aromatic ring vibrations at 1619 and 1533 cm^{-1} [31], nitro group stretching at 1361 cm^{-1} [32], meta-substituted benzene ring deformation at 765 cm^{-1} [33], methoxy stretching at 1280 cm^{-1}, para-substituted benzene vibration at 836 cm^{-1} [34], broad carboxylic O-H stretching (3300–2500 cm^{-1}), carbonyl stretching at 1695 cm^{-1}, and O-H out-of-plane bending at 920 cm^{-1} [34], thereby unambiguously confirming the successful synthesis of high-purity MNBA.

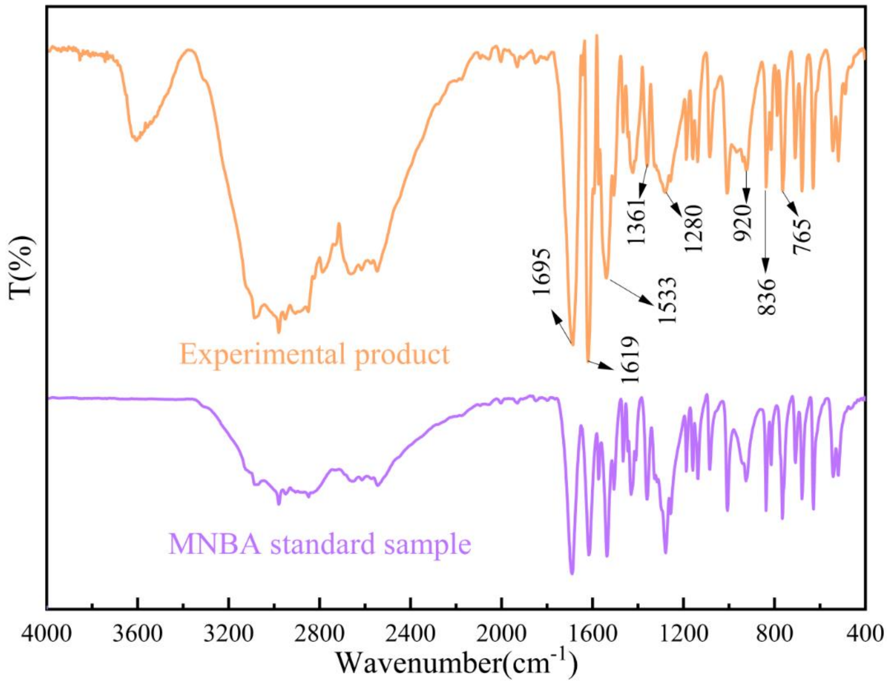

Figure S4. FT-IR spectra of the synthesized product and the MNBA standard.

2.3 Determination of melting point

Melting point determination confirmed the identity and purity of the hydrolysis product, which exhibited a melting range of 66.2–67.2 °C (Table S1), closely matching that of the authentic MMNA standard (67.1–68.1 °C, Table S2). This agreement demonstrates the high purity of the hydrolysis product.

Table S1. Melting point of the hydrolysis product.

Number	1	2	3	4	5	6	Average
Initial (°C)	66.2	65.9	66.3	66.1	66.1	66.4	66.2
Final (°C)	67.2	67.1	67.1	67.3	67.1	67.2	67.2

Table S2. Melting point of the MMNA standard.

Number	1	2	3	4	5	6	Average
Initial (°C)	67.1	66.8	67.3	67.2	67.1	67.2	67.1
Final (°C)	68.1	68.2	68.2	68.1	68.3	67.9	68.1

Melting point determination confirmed the identity and purity of the synthesized product, which exhibited a melting range of 189.1–190.1 °C (Table S3), closely matching that of the authentic MNBA standard (190.0–191.0 °C, Table S4). This agreement demonstrates the high purity of the synthesized compound.

Table S3. Melting point of the synthesized product.

Number	1	2	3	4	5	6	Average
Initial (°C)	189.1	189.0	189.0	189.1	189.2	188.9	189.1
Final (°C)	190.1	190.2	189.9	190.2	190.2	189.9	190.1

Table S4. Melting point of the MNBA standard.

Number	1	2	3	4	5	6	Average
Initial (°C)	189.9	190.1	190.2	189.9	190.0	189.9	190.0
Final (°C)	190.8	191.0	191.1	191.0	190.9	191.2	191.0

2.4 ^{1}H NMR determination and analysis

^{1}H NMR spectroscopy (DMSO-d$_6$, Figure S5) confirmed the identity of the hydrolysis product as MMNA. Characteristic signals included aromatic protons (δ 7.31–7.79, 3H) [35], methylene protons of the hydroxy group (δ 4.49, 2H), and methoxy protons (δ 3.90, 3H) [36]. The chemical shifts, splitting patterns, and integration ratios collectively support the successful formation of the target molecular structure with high purity.

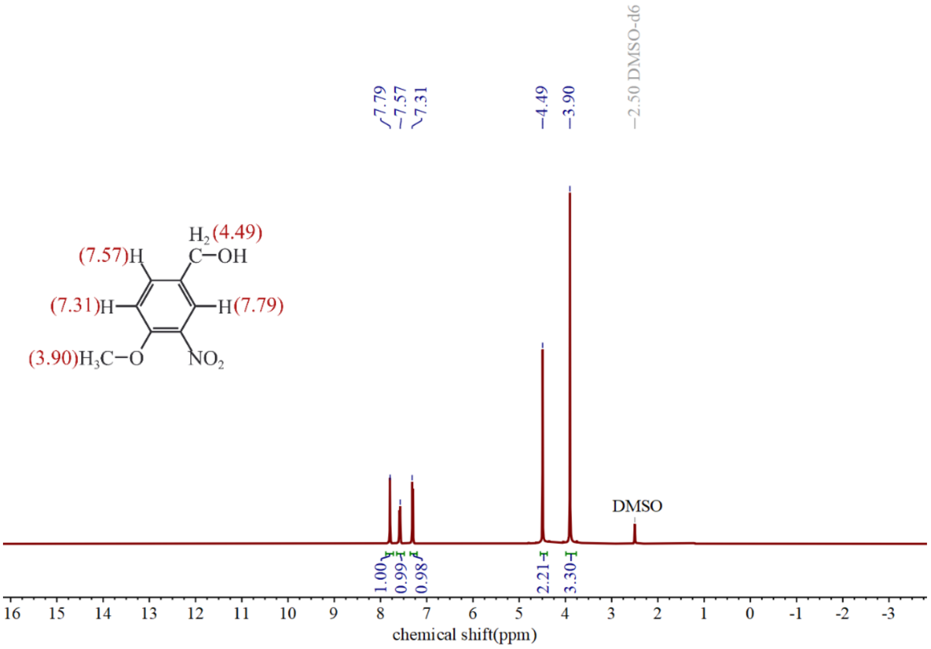

Figure S5. ^{1}H NMR analysis (DMSO-d$_6$) of the hydrolysis product.

^{1}H NMR spectroscopy (DMSO-d$_6$, Figure S6) confirmed the identity of the synthesized product as MNBA. Key resonances included aromatic protons (δ 7.44–8.33, 3H) [35], a carboxylic acid proton (δ 13.30, 1H) [37], and methoxy protons (δ 4.00, 3H) [36]. The agreement of chemical shifts, splitting patterns, and integration ratios with the expected structure confirms the successful synthesis of a high-purity product.

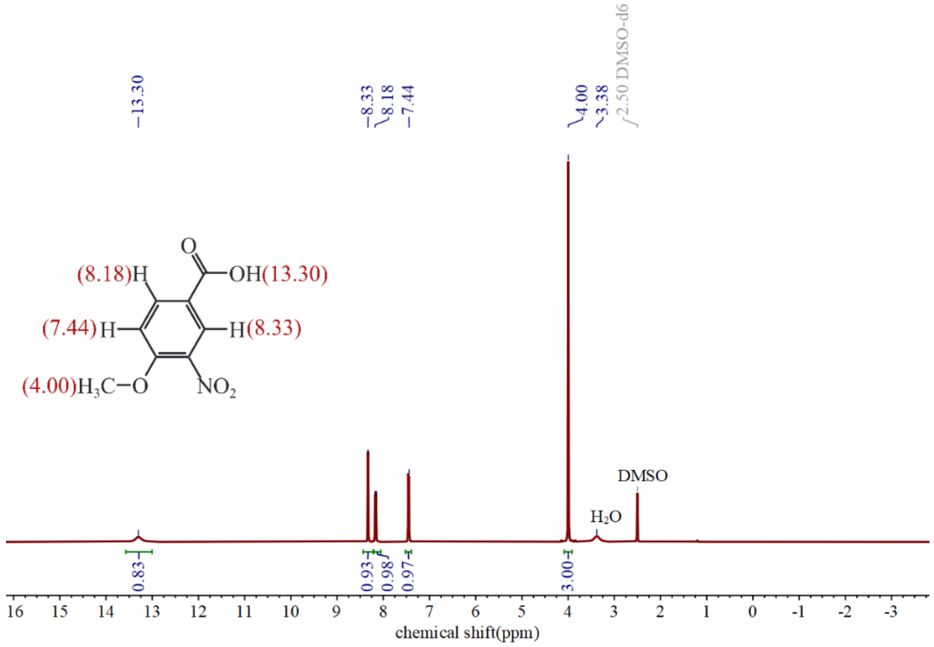

Figure S6. ^{1}H NMR analysis (DMSO-d_6) of the synthesized product.

3. Characterization of intermediate and by-product

3.1 Determination by infrared spectroscopy

The isolated aldehyde intermediate was blended with KBr and compressed into a pellet for Fourier-transform infrared (FT-IR) spectroscopic analysis (Figure S7). The acquired spectrum was then compared to that of an authentic 4-methoxy-3-nitrobenzaldehyde standard, as described in Figure S7. The FT-IR spectrum of the isolated intermediate displayed characteristic absorption bands corresponding to key functional groups: aromatic ring vibrations at 1608 and 1528 cm^{-1} [31], nitro group stretching at 1351 cm^{-1} [32], meta-substitution of the benzene ring at 775 cm^{-1} [32], methoxy stretching at 1278 cm^{-1}, para-substitution at 821 cm^{-1} [33], aldehyde C=O stretching at 1702 cm^{-1}, aldehyde C-H stretching at 2958 and 2871 cm^{-1}[38], and aromatic C-H stretching at 3121 and 3073 cm^{-1} [39]. The close match between the two spectra confirmed the identity of the isolated intermediate as 4-methoxy-3-nitrobenzaldehyde.

The isolated byproduct was mixed with KBr and pressed into a pellet for Fourier-transform infrared (FT-IR) spectroscopic analysis (Figure S8). The obtained spectrum was compared to the standard spectral data of authentic o-nitroanisole reported in the literature [40]. The FT-IR spectrum of the isolated product exhibited characteristic absorption bands corresponding to key functional groups of o-nitroanisole: aromatic sp^2 C–H stretching vibration at 3077 cm^{-1} [40], saturated C–H stretching of the methoxy group (-OCH_3) at 2983 and 2952 cm^{-1} [41], benzene ring skeletal stretching vibrations at 1596 and 1494 cm^{-1}[40], nitro group

(-NO_2) asymmetric stretching at 1524 cm^{-1} and symmetric stretching at 1345 cm^{-1} [41], aromatic C–O–C asymmetric stretching of the methoxy group at 1267 cm^{-1} and symmetric stretching at 1006 cm^{-1}, and ortho-substituted benzene ring C–H out-of-plane bending at 761 cm^{-1} [41]. The close match between the experimental spectrum and the reported standard spectral data of o-nitroanisole confirmed the identity of the isolated byproduct as o-nitroanisole.

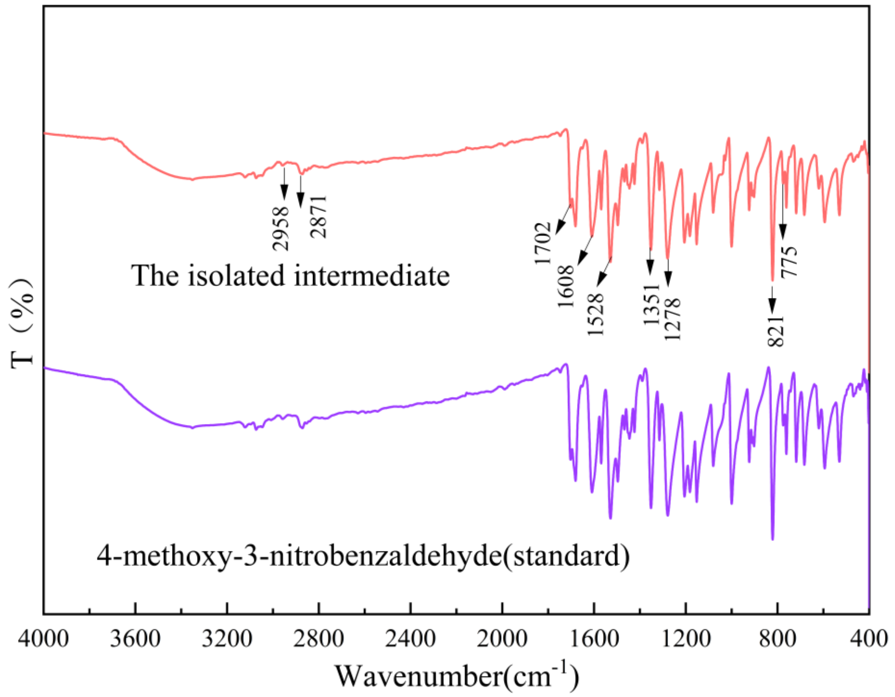

Figure S7. FT-IR spectra of the isolated aldehyde intermediate and the standard.

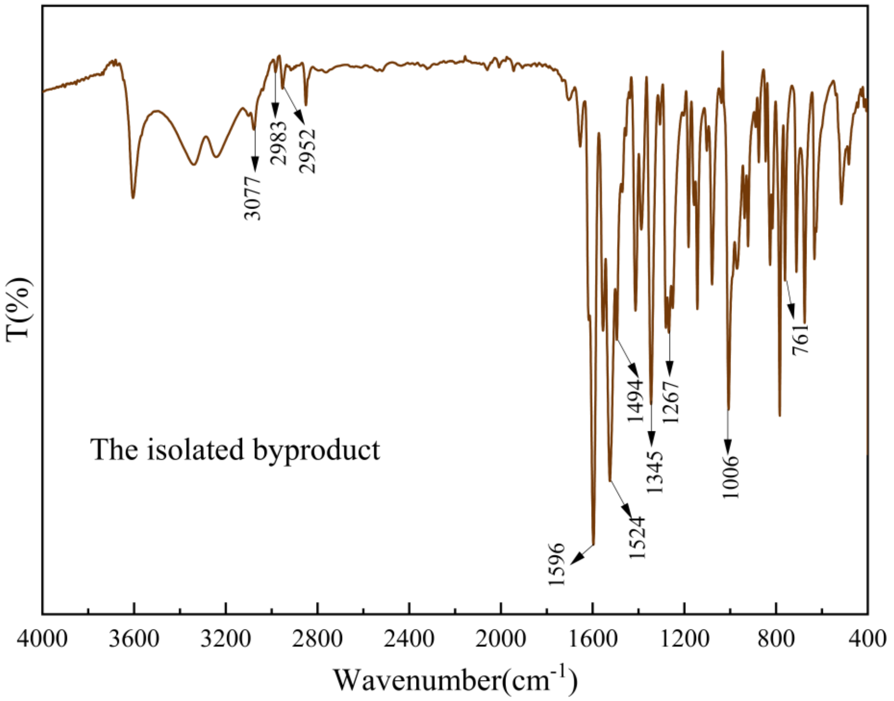

Figure S8. FT-IR spectra of the isolated byproduct.

3.2 ¹H NMR determination and analysis

¹H NMR spectroscopy (DMSO-d_6, Figure S9) confirmed the identity of the isolated intermediate as the desired aldehyde. Characteristic signals included the aldehyde proton (δ 9.95, 1H) [40], aromatic protons (δ 7.59–8.42, 3H) [40], and methoxy protons (δ 4.04, 3H) [40]. The chemical shifts, splitting patterns, and integration ratios collectively support the formation of 4-methoxy-3-nitrobenzaldehyde.

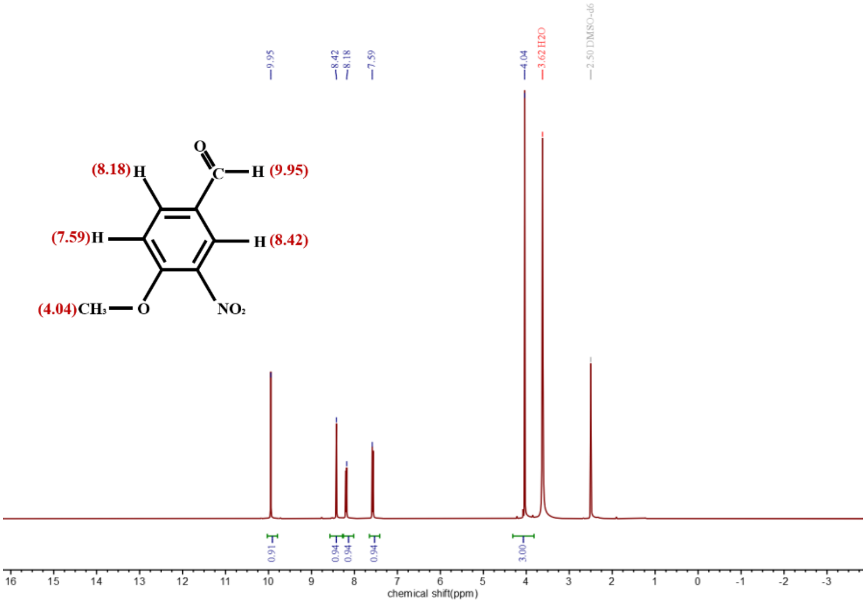

Figure S9. ¹H NMR analysis (DMSO-d_6) of the isolated aldehyde intermediate.

¹H NMR analysis (DMSO-d_6) was performed to determine the isolated byproduct shown in Figure S10. The characteristic signals of aromatic protons (δ 7.25–8.26, 4H) [42] and methoxy group (δ 3.92, 3H) [43] were in good agreement with literature data [40], confirming that the obtained byproduct is o-nitroanisole.

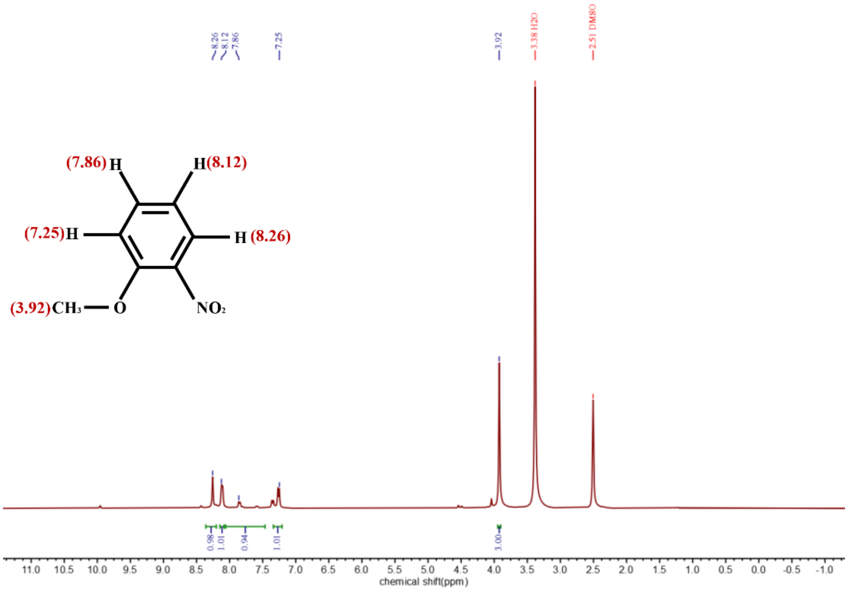

Figure S10. ¹H NMR analysis (DMSO-d_6) of the isolated byproduct.

4.Reaction process monitoring

4.1 Optimized two-step reaction process TLC monitoring

Prior to the reaction, TLC analysis of the reaction mixture revealed that MMNA existed in a relatively stable form (Figure S11).

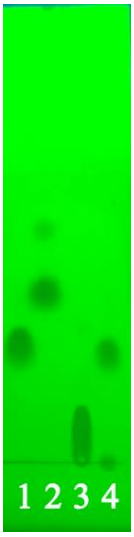

Figure S11. TLC image of optimized two-step reaction mixture.
(1) MMNA (standard), (2) p-Methoxy-m-nitrobenzaldehyde (standard),
(3) MNBA (standard), (4) The reaction mixture.

The reaction system was monitored after addition of sodium hypochlorite for half an hour. TLC analysis indicated that MMNA was thoroughly oxidized within 30 minutes of the reaction, affording p-methoxy-m-nitrobenzaldehyde (Figure S12).

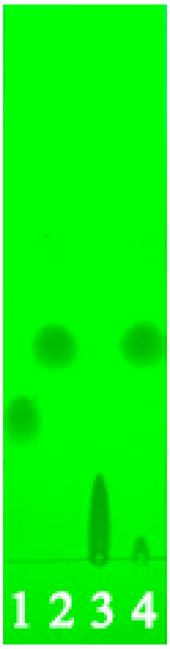

Figure S12. TLC image after the addition of NaOCl for 30 min.
(1) MMNA (standard), (2) p-Methoxy-m-nitrobenzaldehyde (standard),
(3) MNBA (standard), (4) The reaction mixture.

The reaction mixture was monitored after the addition of sodium chlorite after 1 hour. TLC analysis indicated that p-methoxy-m-nitrobenzaldehyde began to be oxidized to MNBA (Figure S13).

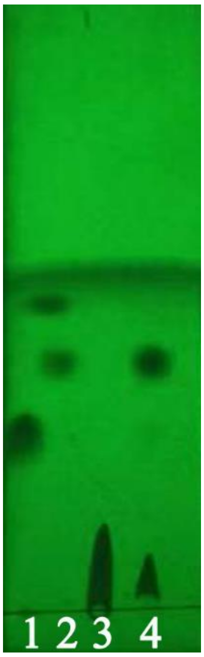

Figure S13. TLC image after the addition of $NaClO_2$ for 1h.
(1) MMNA (standard), (2) p-Methoxy-m-nitrobenzaldehyde (standard),
(3) MNBA (standard), (4) The reaction mixture.

The reaction mixture was monitored after the addition of sodium chlorite for 2 hours. TLC analysis indicated that p-methoxy-m-nitrobenzaldehyde was not yet completely converted (Figure S14).

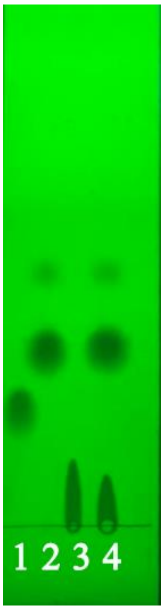

Figure S14. TLC image after the addition of $NaClO_2$ for 2h.
(1) MMNA (standard), (2) p-Methoxy-m-nitrobenzaldehyde (standard),
(3) MNBA (standard), (4) The reaction mixture.

The reaction mixture was monitored after the addition of sodium chlorite for 3 hours. TLC analysis indicated that p-methoxy-m-nitrobenzaldehyde was almost completely converted to MNBA (Figure S15).

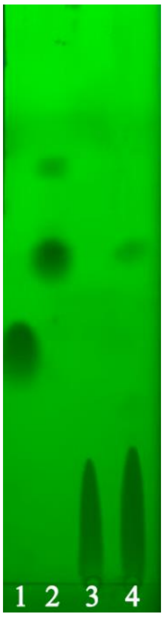

Figure S15. TLC image after the addition of $NaClO_2$ for 3h.

(1) MMNA (standard), (2) p-Methoxy-m-nitrobenzaldehyde (standard),

(3) MNBA (standard), (4) The reaction mixture.

After the addition of sodium chlorite for 4 hours, TLC analysis of the reaction mixture revealed that decarboxylation of MNBA occurred to form o-nitroanisole (confirmed by characterization) (Figure S16).

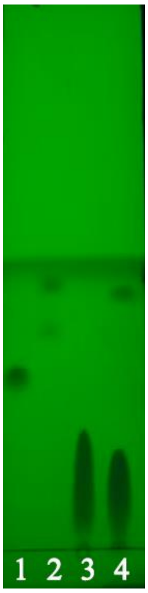

Figure S16. TLC image after the addition of $NaClO_2$ for 4h.

(1) MMNA (standard), (2) p-Methoxy-m-nitrobenzaldehyde (standard),

(3) MNBA (standard), (4) The reaction mixture.

4.2 One-step reaction process TLC monitoring

Prior to the one-step reaction, TLC analysis showed that the MMNA cannot be converted without the addition of oxidant (Figure S17).

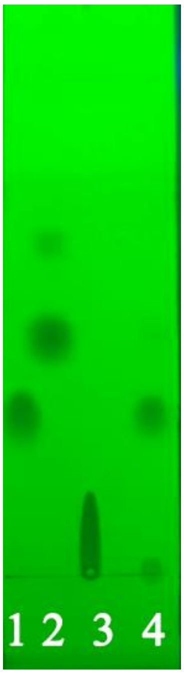

Figure S17. TLC image of one-step reaction mixture.

(1) MMNA (standard), (2) p-Methoxy-m-nitrobenzaldehyde (standard),

(3) MNBA (standard), (4) The reaction mixture.

After 1 hour reaction, TLC analysis revealed that MMNA was almost completely converted, while p-methoxy-m-nitrobenzaldehyde began to undergo oxidation to MNBA (Figure S18).

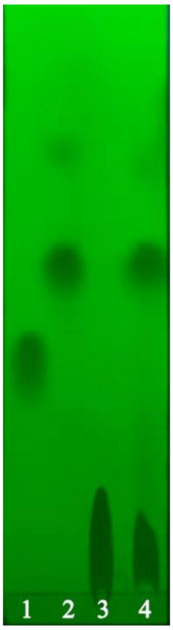

Figure S18. TLC image of the one-step reaction for 1h.

(1) MMNA (standard), (2) p-Methoxy-m-nitrobenzaldehyde (standard),

(3) MNBA (standard), (4) The reaction mixture.

After 2 hours of the one-step reaction, TLC analysis indicated that p-methoxy-m-nitrobenzaldehyde was not completely converted (Figure S19).

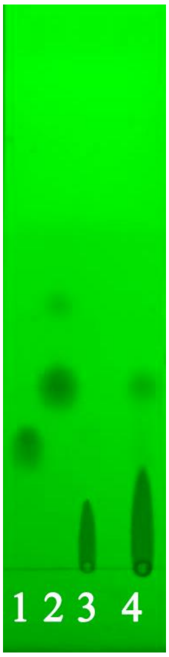

Figure S19. TLC image of the one-step reaction for 2h.

(1) MMNA (standard), (2) p-Methoxy-m-nitrobenzaldehyde (standard),

(3) MNBA (standard), (4) The reaction mixture.

After 3 hours of the one-step reaction, TLC analysis showed that p-methoxy-m-nitrobenzaldehyde was almost completely converted to MNBA (Figure S20).

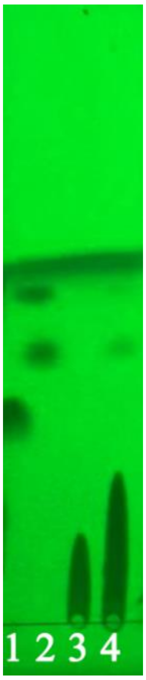

Figure S20. TLC image of the one-step reaction for 3 h.

(1) MMNA (standard), (2) p-Methoxy-m-nitrobenzaldehyde (standard),

(3) MNBA (standard), (4) The reaction mixture.

After 4 hours of the one-step reaction, TLC analysis revealed that further reaction of MNBA occurred to form o-nitroanisole (confirmed by characterization) (Figure S21).

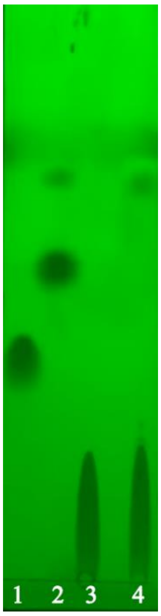

Figure S21. TLC image of the one-step reaction for 4h.

(1) MMNA (standard), (2) p-Methoxy-m-nitrobenzaldehyde (standard),

(3) MNBA (standard), (4) The reaction mixture.
